# Exposure to cigarette smoke affects endometrial maturation including angiogenesis and decidualization

**DOI:** 10.1002/rmb2.12360

**Published:** 2021-01-11

**Authors:** Naoko Kida, Akemi Nishigaki, Maiko Kakita‐Kobayashi, Hiroaki Tsubokura, Yoshiko Hashimoto, Aya Yoshida, Yoji Hisamatsu, Tomoko Tsuzuki‐Nakao, Hiromi Murata, Hidetaka Okada

**Affiliations:** ^1^ Department of Obstetrics and Gynecology Kansai Medical University Osaka Japan

**Keywords:** angiogenesis, cigarette smoking extract, decidualization, endometrial stromal cells, prolactin

## Abstract

**Purpose:**

To elucidate the effects of cigarette smoking on human endometrial maturation for reproductive function, the authors examined the in vitro effects of cigarette smoke extract (CSE) on angiogenesis and decidualization in primary human endometrial stromal cells (ESCs).

**Methods:**

Endometrial stromal cells were cultured with CSE and/or estradiol‐17β (E_2_) and medroxyprogesterone acetate (MPA). The mRNA, protein levels, and protein secretion of the angiogenic factors and decidual specific factors were assessed using real‐time polymerase chain reaction, Western blot analysis, and enzyme‐linked immunosorbent assay, respectively. Decidualization was also monitored by the changes in cellular morphology.

**Results:**

Endometrial stromal cell proliferation substantially decreased after dose‐dependent treatments with CSE at concentrations above 1%, whereas cell death was induced at treatment concentrations above 1% CSE. Treatments above 0.025% CSE led to increased vascular endothelial growth factor mRNA through hypoxia‐inducible factor‐1α accumulation. CSE concentrations at 0.01% and 0.025% increased the prolactin expression levels after treatment with E_2_ and MPA, whereas 0.1% and 0.25% CSE concentrations suppressed prolactin. Similar tendencies were observed in cellular morphology and other decidual specific factors.

**Conclusion:**

These results suggest that exposure to cigarette smoke affects endometrial appropriate maturation including the processes of angiogenesis and decidualization in the reproductive system.

## 1 INTRODUCTION

Smoking and passive smoking affects the establishment and maintenance of pregnancy. The association between tobacco consumption and female infertility in natural cycles is consistently reported in epidemiological studies.^1^ The relationships between ovarian luteal (endocrine) functional factors, endometrial (maternal) factors, and embryo (fetal) factors are important for establishing pregnancy. The effect of smoking on ovarian endocrine function is associated with a lower average age of menopausal women compared to non‐smokers.^2^ Studies on in vitro fertilization cycles have demonstrated that cigarette smoking appears to significantly reduce their ovarian reserve and result in a poor response to ovarian stimulation at an earlier age.^3^ On the other hand, the effect of smoking on the fetal factor is reported to show deleterious changes in the placenta and fetus and is more frequent in pregnant smokers who present higher rates of low birthweight and perinatal and neonatal mortality.^4^ However, as far as we know, the effects of smoking on the human endometrium remain poorly understood.

Angiogenesis also plays a central role in endometrial function, as improper vascularization of the endometrium may cause implantation failure and infertility.^5^, ^6^ Several angiogenic factors in the human endometrium have been identified as important regulators of physiological angiogenesis ^7^, ^8^ including vascular endothelial growth factor (VEGF), stromal cell‐derived factor‐1 (SDF‐1, also known as CXCL12), and angiopoietin 1 and 2 (ANGPT1 and 2).^9^, ^10^, ^11^ VEGF is a key mediator of physiological and pathological vascular remodeling.^12^ SDF‐1 functions as a potent inducer of angiogenesis, stimulating endothelial cell proliferation and cell survival.^13^ ANGPT1 and 2 are a second key group of promotors of angiogenesis and vessel remodeling on the endometrium and interact with VEGF.^14^

The human endometrium undergoes periodic proliferation and differentiation that is controlled during the menstrual cycle by a continuous and careful interaction of the ovarian steroids including estradiol‐17β (E_2_) and progesterone.^15^ Decidualization is an essential process in the differentiation of endometrial stromal cells (ESCs) that is accompanied by dramatic changes in cell function. The decidual reaction plays a central role in embryo implantation and pregnancy establishment.^16^ Many studies have investigated the regulation and the molecular mechanisms involved in decidualization using human‐cultured ESCs in vitro. Morphologically, decidualization is characterized by the transformation of elongated fibroblast‐like ESCs into enlarged round cells with specific structural modifications. The exposure of ESCs to progestin for 12 days, alone or in combination with estrogen, triggers the expression of decidual specific factors such as prolactin (PRL) and insulin‐like growth factor‐binding protein 1(IGFBP‐1). Previously, we demonstrated that progestin increased heart and neural crest derivatives‐expressed transcript 2 (HAND2) expression during ESC decidualization.^17^ HAND2 is a transcription factor required for the growth and development of the heart, branchial arches, and limb buds. Recent reports have demonstrated that progestin‐induced HAND2 regulates decidual specific genes including interleukin‐15 (IL‐15) and fibroblast growth factor 9 (FGF9).^18^, ^19^

In this study, we aimed to clarify the direct effects of smoking on endometrial angiogenesis and decidualization using a human ESCs and a cigarette smoke extract (CSE) an in vitro model.

## 2 MATERIALS AND METHOD

### 2.1 Tissue collection

Human tissues were obtained after written informed consent from each patient in accordance with the Declaration of Helsinki. Human endometrial tissues in the proliferative phase were obtained from 27 women aged 32‐47 years, who underwent hysterectomies due to uterine fibroids. The patients had regular menstrual cycles and no preoperative hormonal treatment. A section of each endometrial specimen was analyzed and confirmed to be histologically normal. This study was approved by the institutional review board of Kansai Medical University, Osaka, Japan (project approval number 2006101).

### 2.2 Cell culture and treatment

ESCs were purified from the endometrial tissues using a standard enzyme digestion method described previously.^20^ ESCs were cultured in DMEM/F‐12 supplemented with 10% fetal calf serum (FCS; Sigma‐Aldrich, St. Louis, MO, USA), 100 IU/mL penicillin, 100 mg/mL streptomycin, and 0.25 μg/mL Amphotericin B (Antibiotic‐Antimycotic 100×; GIbco, Grand Island, NY, USA, MA, USA) at 37°C in a humidified atmosphere of 5% CO_2_. The culture medium was replaced 60 minute after plating to minimize epithelial cell contamination. The percentage of vimentin‐positive cells in the confluent ESCs was confirmed to be >99% according to immunohistochemical staining, as described previously.^21^ After passage 0‐1, when ESCs were nearly confluent, the cells were trypsinized and re‐plated. To negate the effect of endogenous steroid hormones, cells were cultured until confluence and then the medium was replaced with phenol red‐free DMEM/F‐12 supplemented with 10% dextran‐coated charcoal stripped (DCS)‐FCS, Antibiotic‐Antimycotic 1×; Gibco), and 2 mmol/L L‐alanyl‐L‐glutamine (GlutaMAX; Gibco). After 48 hours, ESCs were cultured in DCS‐FCS supplemented with E_2_ (10^−8^ mol/L; Wako, Osaka, Japan) and medroxyprogesterone acetate (MPA; 10^−7^ mol/L; Sigma‐Aldrich) or ethanol as the vehicle control. The ESCs were treated with CSE at various concentrations (0.01%, 0.025%, 0.1, and 0.25%), or untreated, in addition to treatment with ovarian sex steroid hormones.

The culture medium, with or without CSE, was replaced every 3 days for up to 12 days. Experiments using ESCs from each patient were performed at least three times with different cell preparations.

### 2.3 CSE preparations

CSE was prepared as previously described.^22^ The cigarettes used in this study, Mevius (Japan Tobacco, Tokyo, Japan), contained 10 mg of tar and 0.8 mg of nicotine according to the manufacturer's report. Using a constant airflow (0.3 L/min), the smoke of ten consecutive cigarettes was aspirated manually. The smoke was bubbled through 10 mL of phosphate‐buffered saline (PBS) in a 50‐mL polypropylene conical tube. The obtained CSE solution was defined as 100% (one cigarette per 1 mL) and then filtered through a 0.2‐µm filter (ADVANTEC, Tokyo, Japan) to remove bacteria and large particles. Fresh CSE solution was prepared prior to the start of each experiment. After diluting with PBS, the pH of the CSE was between 7.4 and 7.5 for each experiment.

### 2.4 Cell proliferation assay

The proliferation rate of ESCs was detected using a cell counting kit‐8 (CCK‐8; Dojindo, Kumamoto, Japan) according to the manufacturer's instructions. In brief, ESCs were seeded in 96‐well plates at a density of 5 × 10^3^ cells per well. After culturing for 24 hours, cells were treated with increasing concentrations of CSE (0, 0.01%, 0.025%, 0.1%, 0.25%, 0.5%, 1%, 2%, or 3%) for 24 hours. CCK‐8 stain was subsequently added, and the absorbance (optical density) at 450 nm was detected using a microplate reader (EnSpire; PerkinElmer, Inc, Yokohama, Japan). Each experiment was conducted in triplicate.

### 2.5 Cytotoxicity lactase dehydrogenase assay

The level of lactase dehydrogenase (LDH) released was determined using cytotoxicity LDH assay kit‐WST (Dojindo, Kumamoto, Japan) to examine the effect of CSE treatment on cell viability. ESCs were seeded in 96‐well plates at a density of 5 × 10^3^ cells per well. After exposure to various concentrations of CSE for 24 hours, the working solution was subsequently added according to the manufacturer's instructions and the absorbance (optical density) at 490 nm was determined using a microplate reader (EnSpire; PerkinElmer, Inc). Each experiment was conducted in triplicate.

### 2.6 Cell death detection via enzyme‐linked immunosorbent assay

Apoptosis of ESCs was quantified by directly determining the extent of nucleosomal DNA fragmentation via cell death detection enzyme‐linked immunosorbent assay (ELISA) according to the manufacturer's protocol (Roche Diagnostic GmbH, Mannheim, Germany). Briefly, cell lysates were obtained from ESCs treated with various concentrations of CSE for 24 hours. The substrate solution was added to each well, and the absorbance was read at 405 and 490 nm as a reference.

### 2.7 Semi‐quantitative real‐time polymerase chain reaction

Total RNA was isolated from cultured ESCs using the RNeasy Minikit (Qiagen, Hilden, Germany) according to the manufacturer's instructions. The first‐strand cDNA synthesis kit ReverTra Ace qPCR RT Master Mix (Toyobo, Osaka, Japan) was used for cDNA synthesis. Reverse transcription was performed according to the manufacturer's instructions. Real‐time PCR (RT‐PCR) was performed using the Rotor‐Gene Q HRM (Qiagen) and the Thunderbird SYBR qPCR Mix kit (Toyobo), according to the manufacturer's instructions. The PCR efficiency (E%) for the amplification of each gene was calculated using the following formula: E% = [−1 + 10(−1/α)] × 100, where α is the slope of the corresponding amplification plot.^23^ For relative quantification, data were normalized against elongation factor‐1α (*EF‐1α*) as an internal control. The validated primer sequences are listed in Table 1.

**TABLE 1:** Primer sequences used for real‐time PCR

Target	Gene symbol	Forward primer 5′‐3′	Reverse primer 5′‐3′
*VEGF*	VEGFA	CGAAACCATGAACTTTCTGC	CCTCAGTGGGCACACACTCC
*GLUT1*	SLC2A1	TCCACGAGCATCTTCGAGA	ATACTGGAAGCACATGCCC
*ANGPT1*	ANGPT1	CAGCGCCGAAGTCCAGAAAAC	CACATGTTCCAGATGTTGAAG
*ANGPT2*	ANGPT2	AACATCCCAGTCCACCTGAG	GGTCTTGCTTTGGTCCGTTA
*SDF‐1*	CXCL12	TCGTGCTGACCGCGCTCTGCCTCA	TCTGAAGGGCACAGTTTGGAGTGT
*PRL*	PRL	ATTCGATAAACGGTATACCCATGGC	TTGCTCCTCAATCTCTACAGCTTTG
*IGFBP‐1*	IGFBP1	CTATGATGGCTCGAAGGCTC	TTCTTGTTGCAGTTTGGCAG
*HAND2*	HAND2	AGAGGAAGAAGGAGCTGAACGA	CGTCCGGCCTTTGGTTTT
*IL‐15*	IL15	GTTCACCCCAGTTGCAAAGT	CCTCCAGTTCCTCACATTC
*FGF9*	FGF9	GGGGAGCTGTATGGATCAGA	TTCCAGTGTCCACGTGCTTA
*EF1α*	EEF1A1	TCTGGTTGGAATGGTGACAACATGC	AGAGCTTCACTCAAAGCTTCATGG

### 2.8 Western blot analysis

Whole cell lysates were prepared using lysis buffer containing mammalian protein extraction reagent (Thermo Fisher Scientific Inc, Rockford, IL, USA) and a protease inhibitor cocktail (Calbiochem, La Jolla, CA, USA). Samples were centrifuged at 10 000 × g to pellet the cell debris. The protein concentrations were quantified using Bio‐Rad protein assay reagent (Bio‐Rad Lab., Hercules, CA, USA). Equivalent amounts of lysate protein (20 μg/lane) were electrophoresed on a 7.5% sodium dodecyl sulfate‐polyacrylamide gel electrophoresis and electro‐transferred onto membranes using the Trans‐Blot Turbo^™^ Transfer System (Bio‐Rad, Laboratories, Inc). Non‐specific‐binding sites were blocked with Blocking One solution (Nacalai tesque, Kyoto, Japan) for 20 minutes. The membranes were probed with purified mouse anti‐human HIF‐1α antibody (1:1000; BD Transduction Laboratories, Tokyo, Japan) and mouse monoclonal β‐actin antibody (1:5000; Sigma‐Aldrich) as the primary antibody, and anti‐mouse IgG peroxidase‐labeled secondary antibody (1:5000; GE Healthcare Life Science) as the secondary antibody. Immune complexes were visualized using enhanced chemiluminescence plus Western blotting detection reagents (GE, Little chalfont, UK). Experiments were performed in triplicate, and representative blots are shown.

### 2.9 Determining VEGF and PRL levels using ELISA

The levels of VEGF and PRL released in cell culture supernatants were determined using a commercially available ELISA kit (Duoset^®^ ELISA kit; R&D Systems, Minneapolis, MN, USA). Intra‐ and inter‐assay coefficients of variation in cell culture supernatants were 4.7% and 5.5% for VEGF and 2.2% and 8.9% for PRL, respectively.

### 2.10 Morphological analysis of decidualized ESCs

For morphological assessments, each sample was stained using the May‐Grunwald Giemsa staining technique and a Diff‐Quik kit (Sysmex, Hyogo, Japan) according to the manufacturer's instructions. Trypsinized ESCs were re‐plated on Lab‐Tek chamber slides (Thermo Fisher Scientific, Waltham, MA, USA) prior to cell staining. Morphological changes associated with decidualization were observed under a microscope (E1000M; Nikon, Tokyo, Japan). The shape index (SI) was determined to evaluate the circularity of cells and nuclei using the ImageJ software. SI is calculated using the following formula: SI = 4π × area/perimeter^2^, which is an established method originally reported to determine vascular cell shape.^24^ A circle would have an SI of 1, whereas a straight line would have an SI of 0. The cell area was also calculated. SI and cell area values were used to quantitate the cell and nuclear shape changes associated with decidualization in 10 samples.^25^ Cell SI was determined for low‐power field (×100) images, whereas nuclear SI was determined for high‐power field (×100) images.

### 2.11 Statistical analysis

Data are presented as the mean ± standard deviation. Statistical analyses were performed using JMP 12 software. Groups were compared using one‐way analysis of variance (ANOVA) followed by Dunnett's test for multiple comparisons. Analysis of two independent factors was performed using the two‐way ANOVA. The Tukey‐Kramer test was used to compare the means between groups in which statistically significant differences were found. A value of *P* < .05 was considered statistically significant.

## 3 RESULTS

### 3.1 Effects of CSE on cell proliferation, cytotoxicity, and apoptosis

**FIGURE 1:**
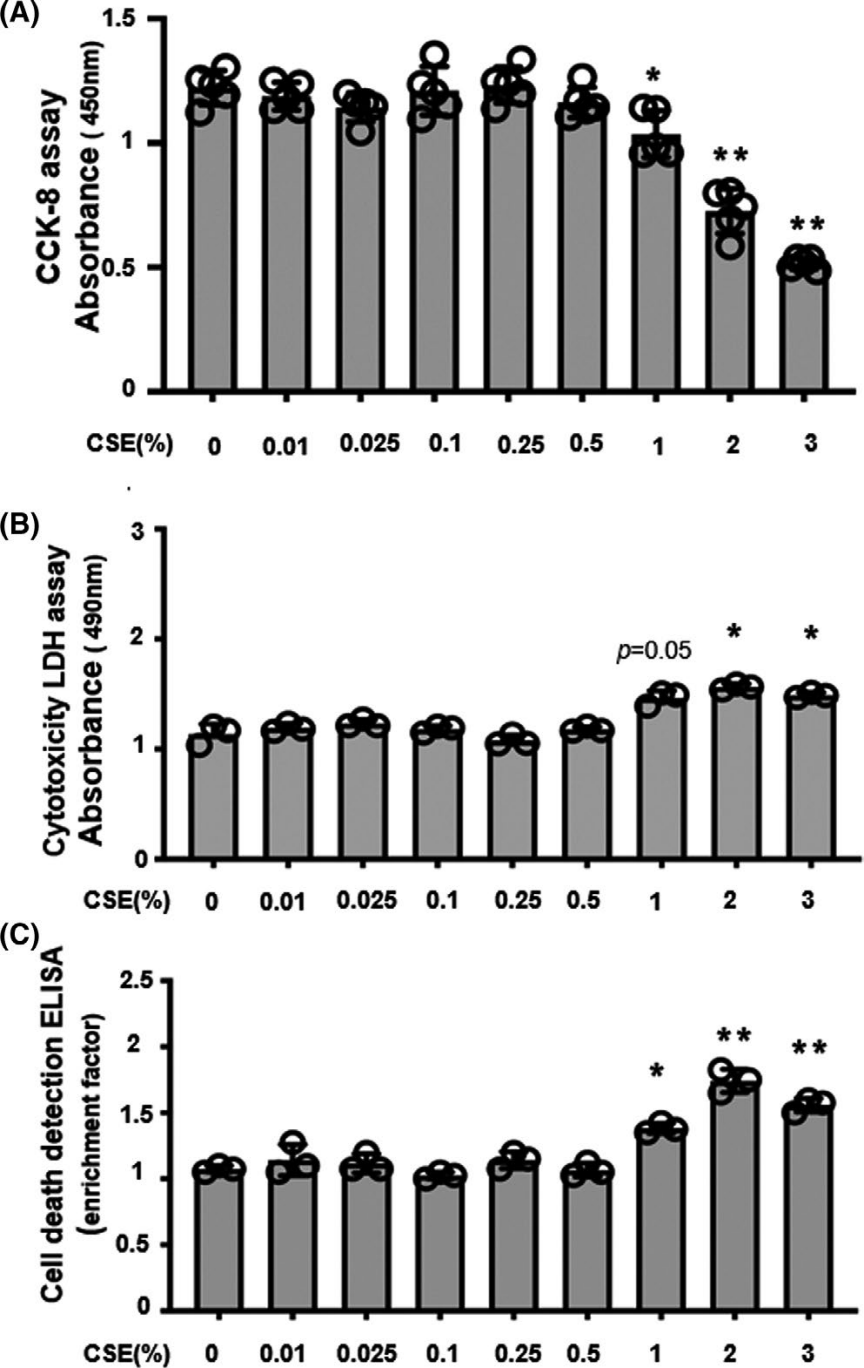
Dose‐dependent effect of cigarette smoke extract (CSE) on (A) cell proliferation, (B) cytotoxicity, and (C) apoptosis Human endometrial stromal cells (ESCs) were cultured for 24 h in medium containing CSE (0.01% to 3%) or without treatment (control 0%). ESCs were subsequently incubated with cell counting kit 8 solution for 2 h, and absorbance was measured at 450 nm (A). Lactate dehydrogenase toxicity was measured using the Cytotoxicity LDH Assay Kit‐WST (B). Cell apoptosis was determined via cell death detection enzyme‐linked immunosorbent assay (C). Data are presented as the mean ± SD for combined data of at least three separate experiments with different cell preparations. Statistically significant differences are indicated as *P < .05, **P < .01 vs control (CSE 0%)

To investigate the proliferative, cytotoxic, and apoptotic effects of CSE on ESCs, the cells were cultured with various concentrations of CSE for 24 hours. As shown in Figure 1A, up to 0.5% CSE had no effect on the cells; however, a significant dose‐dependent inhibition of ESC proliferation was observed at treatment concentrations of >1% CSE. As shown in Figure 1B, up to 0.5% CSE had no cytotoxic effects whereas significant cytotoxicity was seen above 2%, and cytotoxicity trend observed at 1% CSE (*P* = .05). As shown in Figure 1C, apoptosis was observed at treatment concentrations above 1% CSE. Therefore, for subsequent experiments, concentrations of CSE below 0.5% were used.

### 3.2 Effect of angiogenic factors on mRNA expression

**FIGURE 2:**
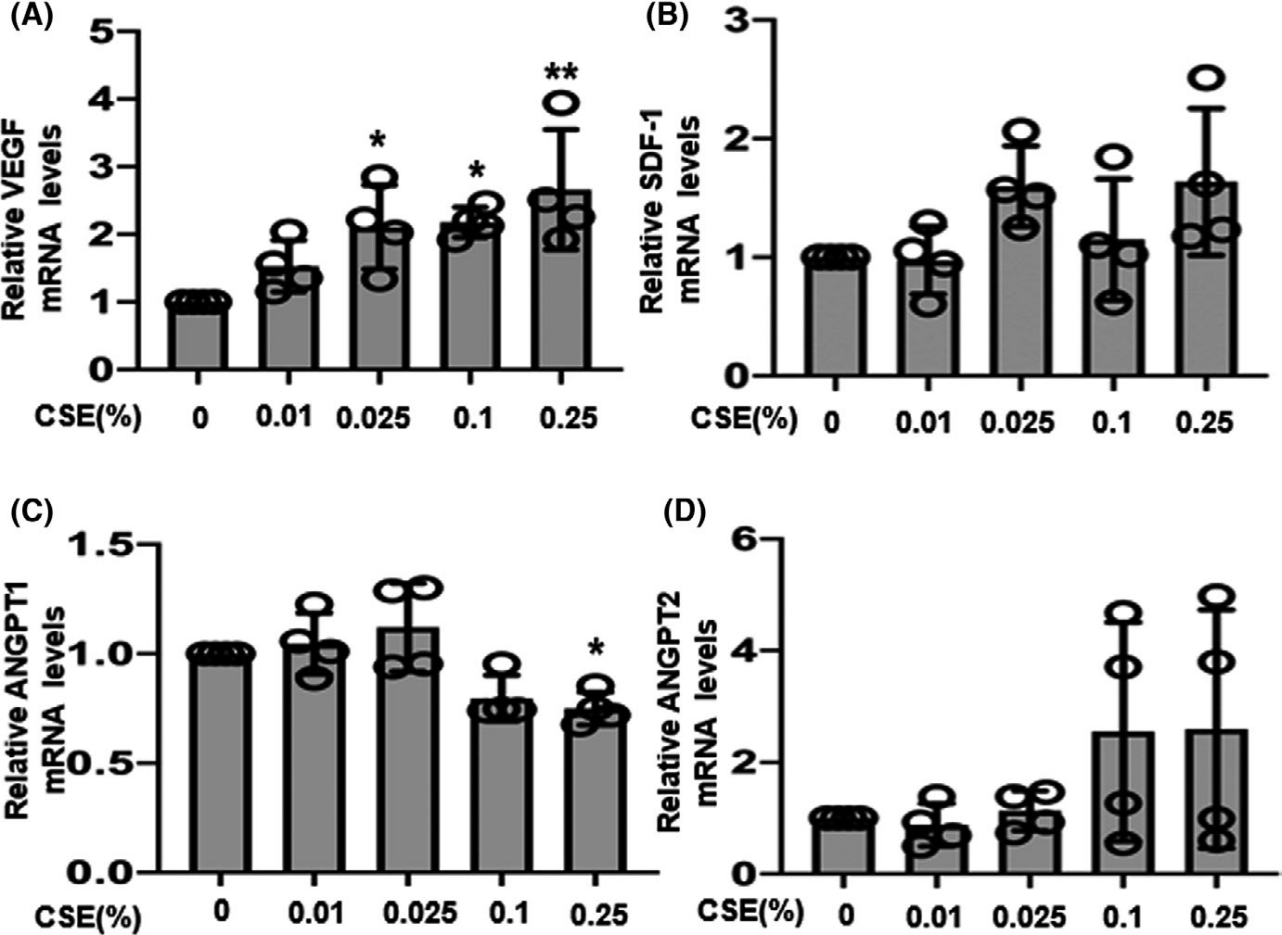
Effects of cigarette smoke extract (CSE) on mRNA expression of the angiogenic factors cells were incubated with medium containing CSE at 0.01%, 0.025%, 0.1%, and 0.25% concentrations, or medium alone (control 0%) for 12 d. The mRNA levels of (A) vascular endothelial growth factor (*VEGF*), (B) stromal cell‐derived factor‐1 (*SDF‐1*), (C) angiopoietin 1 and 2 (*ANGPT1*), and (D) *ANGPT2* were assessed by quantitative real‐time polymerase chain reaction and calculated after normalization to *EF1α* mRNA levels. Data are presented as the mean ± SD, n = 4. Statistically significant differences are indicated as *P < .05, **P < .01 vs control (CSE 0%)

The effect of CSE on the mRNA expression of angiogenic factors was evaluated. As shown in Figure 2A, 0.01% CSE had no effect on *VEGF* mRNA expression compared to that in control cells, whereas a significant increase was observed at concentrations above 0.025%. CSE did not affect *SDF‐1* mRNA expression at any of the CSE concentrations tested (Figure 2B). As shown in Figure 2C, 0.25% CSE significantly decreased the expression of *ANGPT1* mRNA, whereas no significant changes in *ANPGT2* mRNA expression were observed at any concentration (Figure 2D). Of the known angiogenic factors expressed in the human endometrium, the expression of only *VEGF* was significantly induced by CSE in a dose‐dependent manner.

### 3.3 Effects of CSE on the time course of VEGF secretion

**FIGURE 3:**
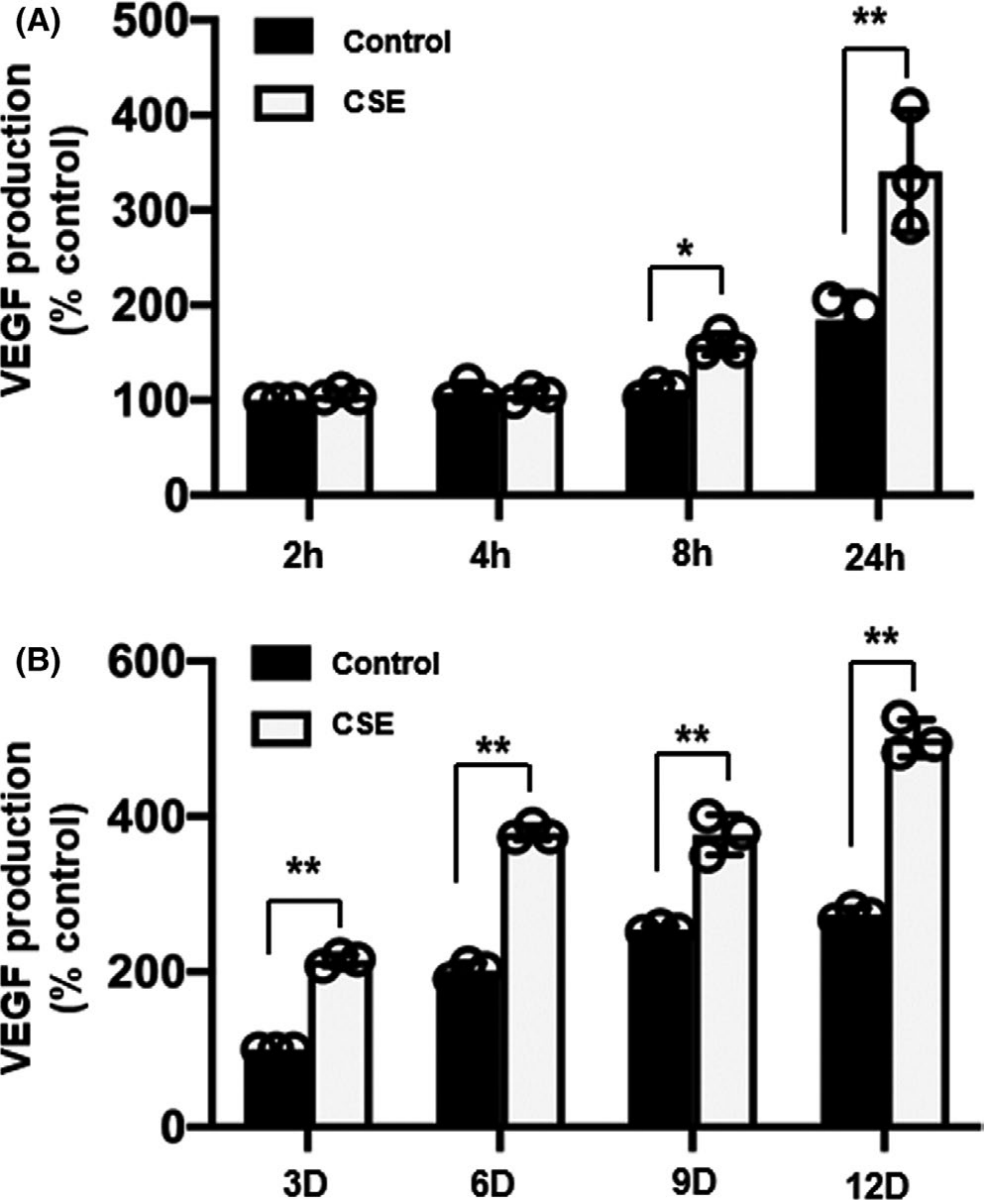
Effects of cigarette smoke extract (CSE) on time course of vascular endothelial growth factor (VEGF) secretion endometrial stromal cells were cultured with either control or 0.25% CSE for 2, 4, 8 and 24 h (A) or 3, 6, 9, and 12 d (B). The cell lysates were analyzed using enzyme‐linked immunosorbent assay. Data are presented as the mean ± SD, n = 3. Statistically significant differences are indicated as *P < .05, **P < .01 vs control (CSE 0%)

To investigate the effects of CSE on the time course of VEGF secretion, ESCs were cultured with or without 0.25% CSE for various periods of time. A 0.25% concentration of CSE enhanced VEGF levels in a time‐dependent manner (Figure 3A,B). A 0.25% concentration of CSE caused a significant increase in VEGF production after 8 hours of culture compared with that in the control, and this increase continued until 12 days.

### 3.4 Effect of CSE in HIF‐1α protein and glucose transporter 1 mRNA expression

**FIGURE 4:**
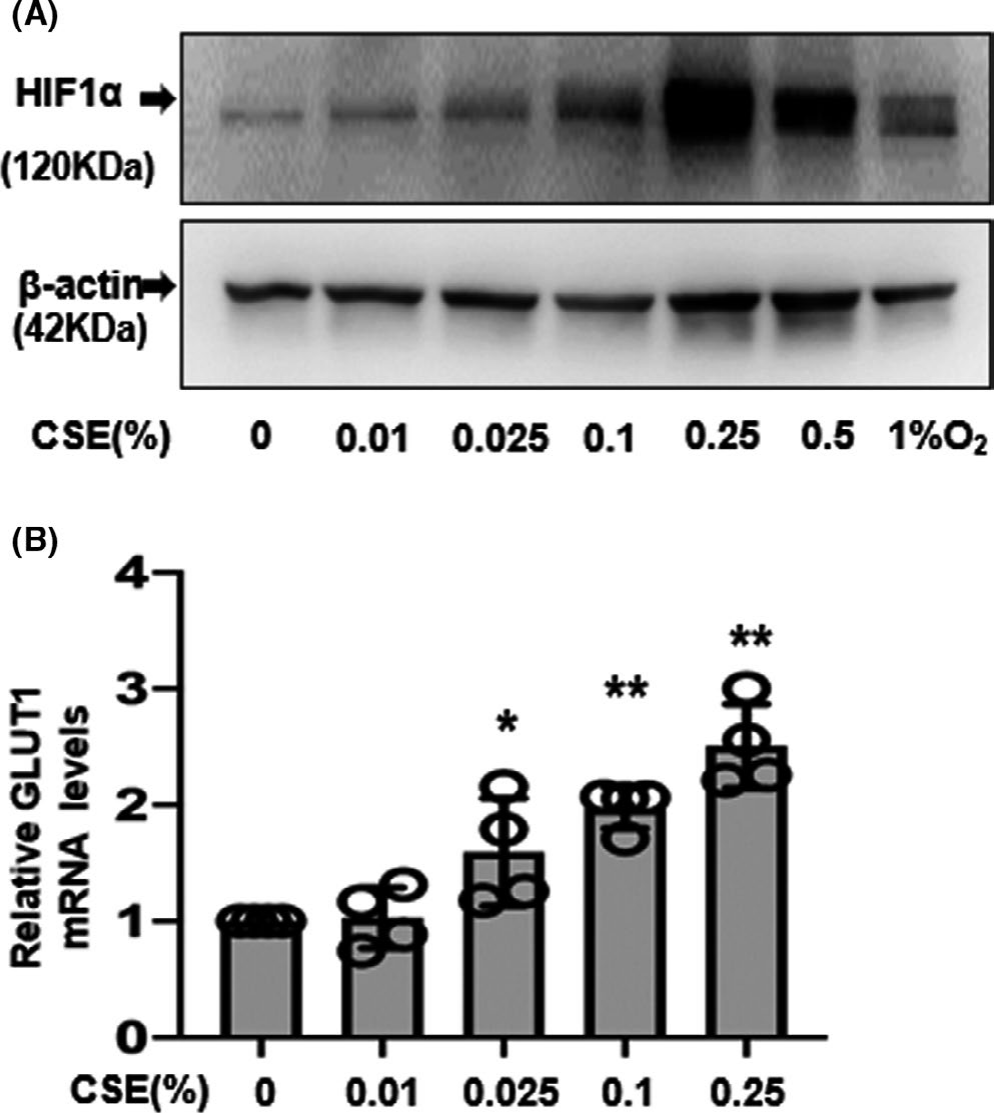
Effect of cigarette smoke extract (CSE) on HIF‐1α protein and GLUT1 mRNA expression (A) endometrial stromal cells were incubated for 4 h in medium containing CSE (0.01%, 0.025%, 0.1%, 0.25%, and 0.5%) or medium alone (0%). The expression of HIF‐1α was quantified by Western blotting. (B) *GLUT1* mRNA levels were assessed by quantitative real‐time polymerase chain reaction and calculated after normalization to *EF1α* mRNA levels. Data are presented as the mean ± SD, n = 4. Statistically significant differences are indicated as *P < .05, **P < .01 vs control (CSE 0%)

It has been demonstrated that *VEGF* mRNA expression increases as a downstream target gene of HIF‐1α in ESCs.^26^ To investigate the effect of CSE on HIF‐1, ESCs were exposed to CSE at various concentrations under normoxic (20% O_2_) or hypoxic (1% O_2_) conditions for 4 hours. As shown in Figure 4A, CSE induced the accumulation of HIF‐1α protein in a dose‐dependent manner. The highest accumulation of HIF‐1α protein was observed after treatment with 0.25% CSE, while the levels had attenuated with 0.5% CSE. These results suggest that CSE promotes the accumulation of HIF‐1α to a similar extent as 1% O_2_. We then evaluated the mRNA expression of glucose transporter 1 (*GLUT1*), which is one of the important downstream genes of HIF‐1α, as is *VEGF*.^27^ Results showed a significant increase in the *GLUT1* mRNA expression after culturing with concentrations above 0.025% CSE (Figure 4B). These findings indicate that HIF‐1α may be responsible for the CSE‐induced *VEGF* and *GLUT1* expression.

### 3.5 Effects of CSE on decidualization

**FIGURE 5:**
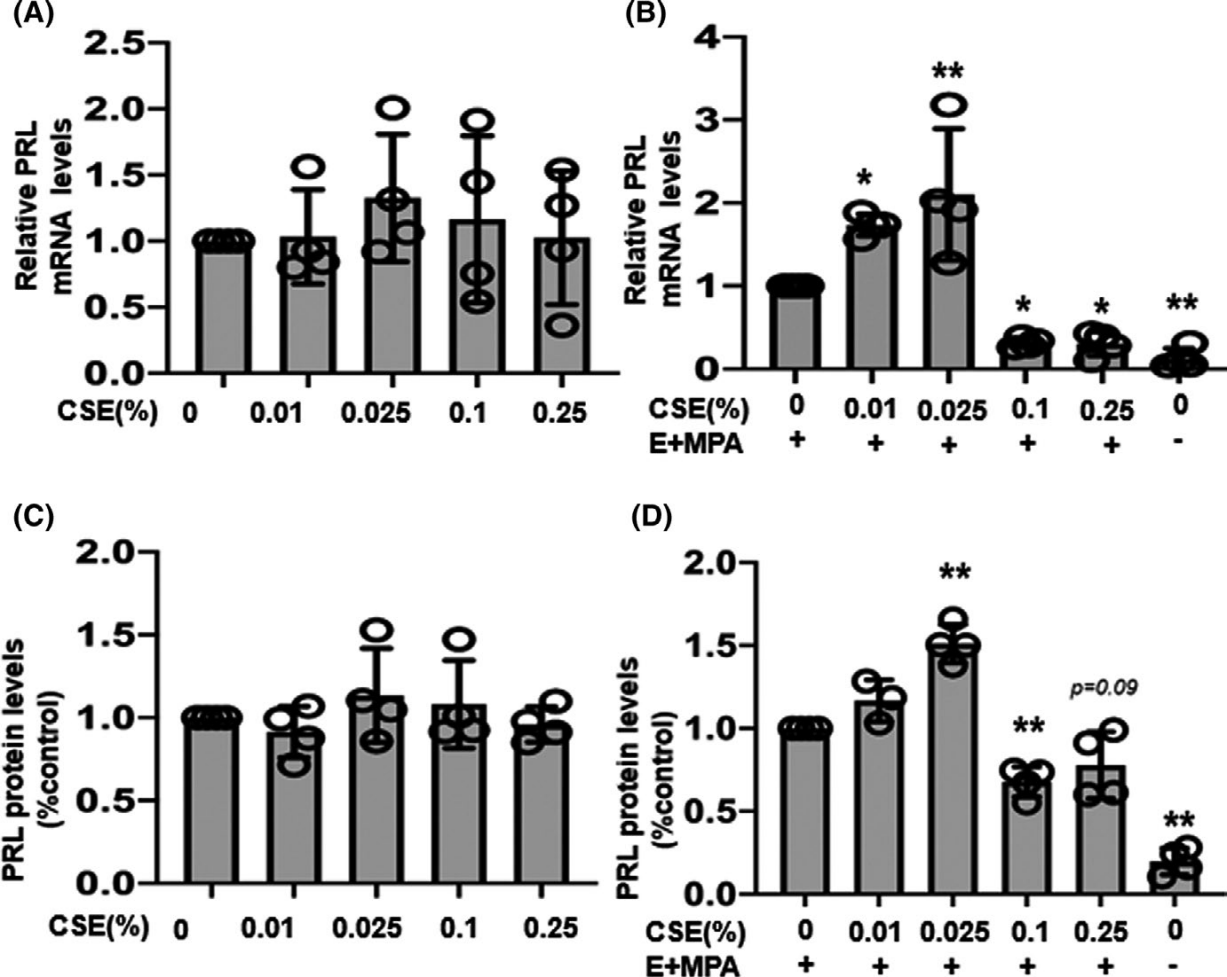
Effects of cigarette smoke extract (CSE) on prolactin (PRL) mRNA expression and production. Human endometrial stromal cells (ESCs) were treated with CSE (0%, 0.01%, 0.025%, 0.1%, and 0.25%) and/or E_2_ (10^−8^ mol/L) + MPA (10^−7^ mol/L) for up to 12 d. A,B, *PRL* mRNA expressions were assessed via quantitative real‐time polymerase chain reaction and calculated after normalization to *EF1α* mRNA levels. C,D, PRL production in the culture supernatants treated with E_2_ + MPA for 12 d was analyzed using enzyme‐linked immunosorbent assay. Data are presented as the mean ± SD, n = 4. Statistically significant differences are indicated as *P < .05, **P < .01 vs control (CSE 0%)

To investigate the effect of CSE on decidualization, the expression of PRL, which is a specific decidual marker, was analyzed. Cultured ESCs were treated with medium containing varying concentrations of CSE, with or without the ovarian hormones E_2_ and MPA. In the treatment conditions without E_2_ and MPA, no significant differences in *PRL* mRNA (Figure 5A) and PRL protein expression (Figure 5C) were observed following treatment with any of the CSE concentrations tested, compared to control. In contrast, in the presence of E_2_ and MPA, the levels of *PRL* mRNA and PRL protein were elevated after treatment with 0.01% and 0.025% CSE compared to control, but expression levels were then suppressed at 0.1% and 0.25% CSE concentrations (Figure 5B,D).

### 3.6 Effects of CSE on cell morphology

**FIGURE 6:**
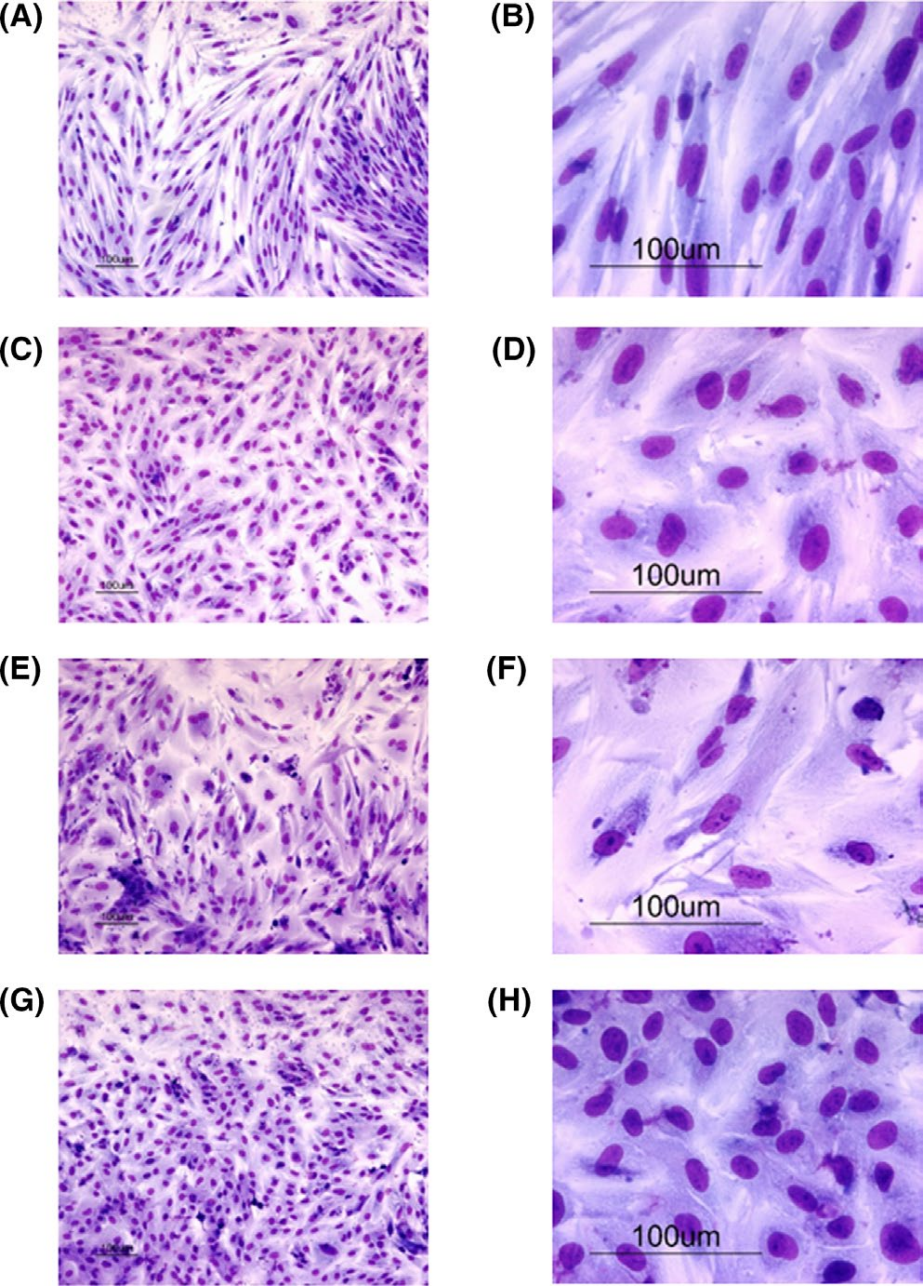
Effects of cigarette smoke extract (CSE) on cell morphology during decidualization. Human endometrial stromal cells (ESCs) were cultured with the following agents for 12 d: (A,B) vehicle (control); (C,D) E_2_ (10^−8^ mol/L) + MPA (10^−7^ mol/L); (E and F) E_2_ (10^−8^ mol/L) + MPA (10^−7^ mol/L) + 0.01% of CSE; (G and H) E_2_ (10^−8^ mol/L) + MPA (10^−7^ mol/L) + 0.1% of CSE. (A, C, E, and G): low‐power field (×100) images. (B, D, F, and H): high‐power (×400) images. The scale line represents 100 μm

**FIGURE 7:**
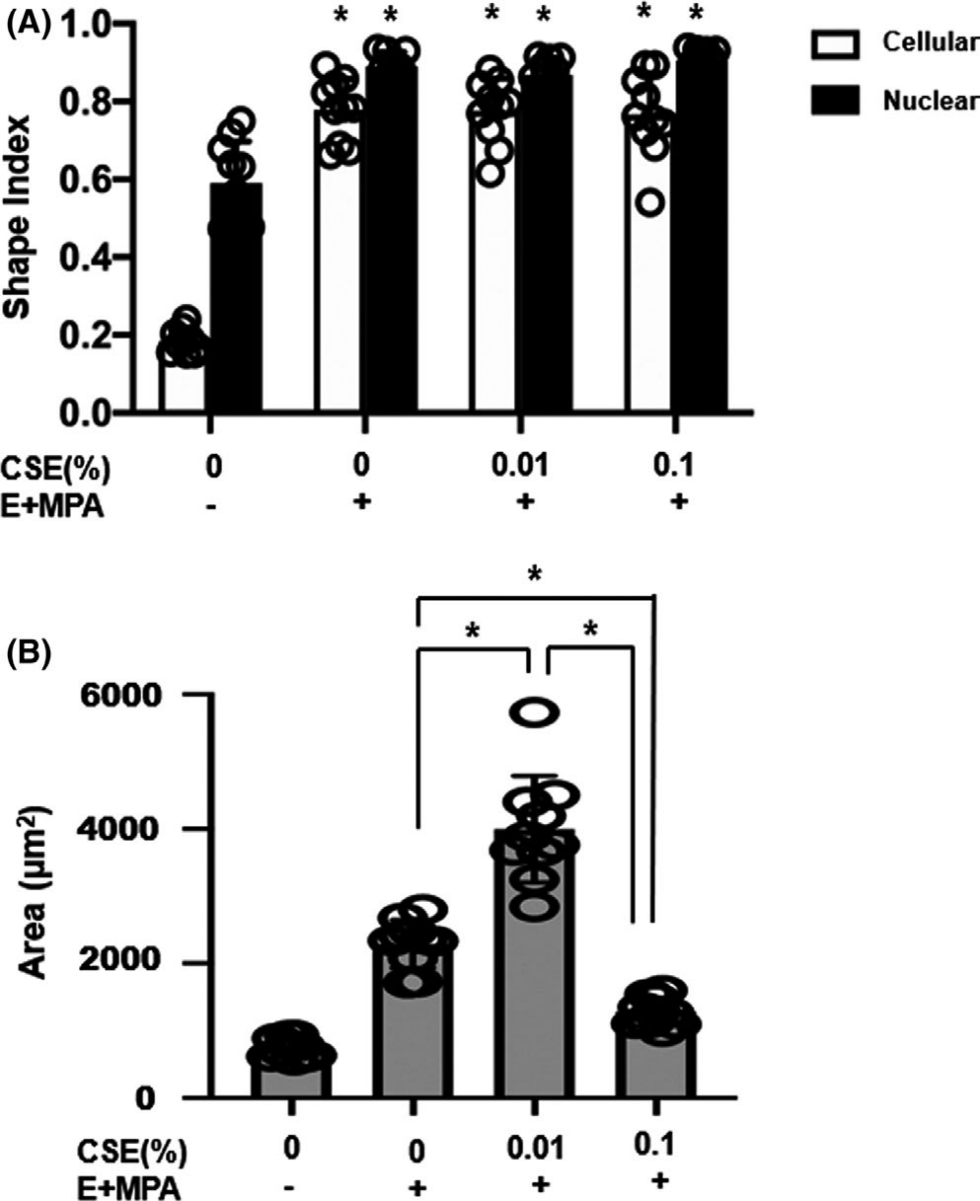
The shape index (SI) of circularity and cell area in endometrial stromal cells during decidualization with or without cigarette smoke extract (CSE) treatment. A, In order to quantify morphological differences, cellular and nuclear shape index were calculated from each condition in Figure 6 (n = 10). B, The cell areas were calculated from each condition in Figure 6 (n = 10). Each value represents mean ± SD; *P < .01 vs control (CSE 0% without E + MPA treatment)

A defining feature of decidualization is the morphological change induced in ESCs, which includes the development of a round shape and cobblestone morphology with a concomitant increase in cell area.^25^ To examine the effect of CSE on cell morphology, ESCs were treated with ovarian hormones E_2_ and MPA for 12 days and morphological changes were evaluated. The SI of circularity and cell area was also determined to quantify the changes in cell morphology. The all‐decidualized ESCs with or without CSE showed that the characteristic morphology of the elongated spindle‐shaped cells changed into enlarged polygonal cells and a statistically significant increase in the SI of the cells and nuclei was observed compared to the ESCs treated without E_2_ and MPA (Figure 7A). Treatment with 0.01% CSE combined with E_2_ and MPA further enlarged the cell area than the E_2_ and MPA treatment alone (Figure 6E,F and 7B). In contrast, at 0.1% concentration of CSE with E_2_ and MPA, cell area decreased compared to that of ESCs treated with E_2_ and MPA alone (Figure 6G,H, and 7B). These results indicated that the characteristic morphological changes were enhanced at 0.01% CSE and attenuated at 0.1% CSE, thus confirming the ability of CSE to affect decidualization.

### 3.7 Effects of CSE on mRNA levels of decidual specific factors

**FIGURE 8:**
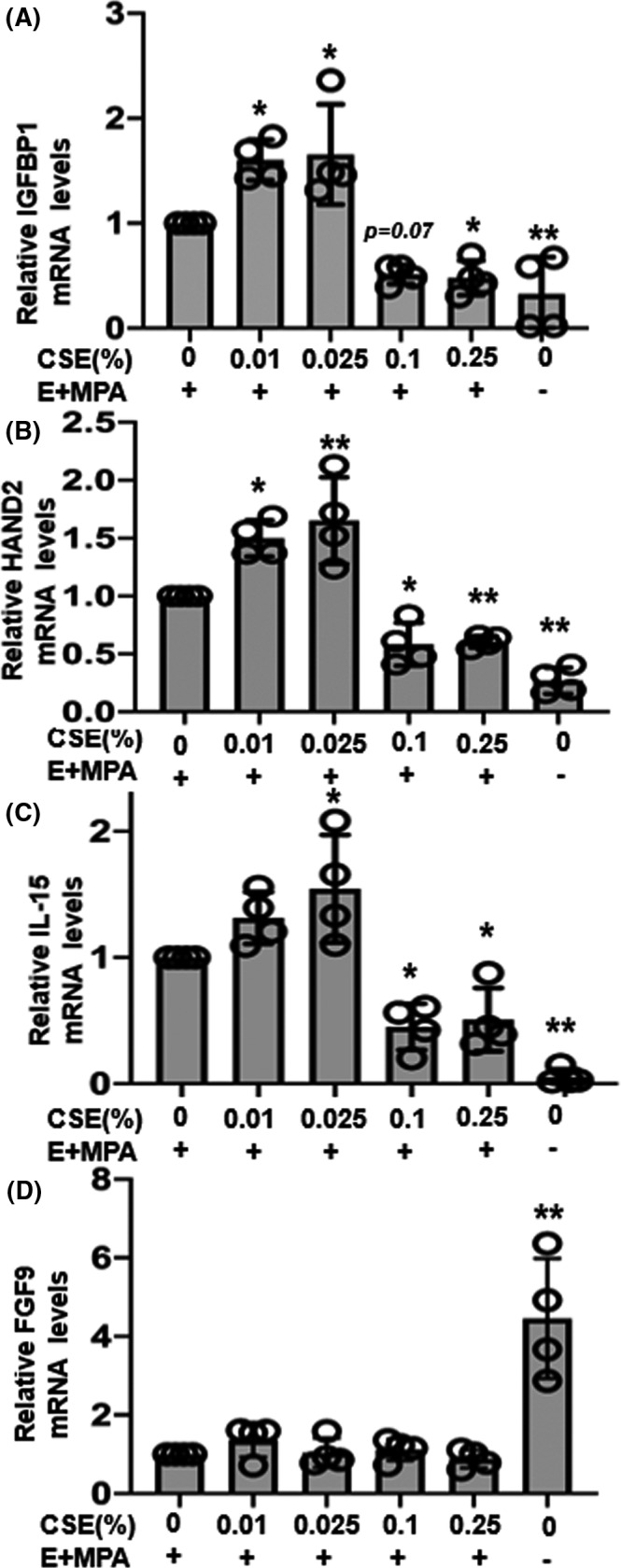
Effects of cigarette smoke extract (CSE) on mRNA levels of decidual specific factors. Human endometrial stromal cells (ESCs) were treated with CSE (0%, 0.01%, 0.025%, 0.1%, and 0.25%) and/or E_2_ (10^−8^ mol/L) + MPA (10^−7^ mol/L) for up to 12 d. The mRNA expression of (A) insulin‐like growth factor‐binding protein 1 (*IGFBP‐1*), (B) heart and neural crest derivatives‐expressed transcript 2 (*HAND2*), (C) interleukin 15 (*IL‐15*), and (D) fibroblast growth factor 9 (*FGF9*) were analyzed via RT‐PCR. Data were normalized to the *EF‐1α* housekeeping gene and are shown as the mean ± SD (n = 4). *P < .05, **P < .01 vs control (CSE 0%)

The effects of CSE on decidual specific factors, including IGFBP‐1, HAND2, IL‐15, and FGF9, were evaluated. ESCs treated with varying concentrations of CSE showed no significant differences in the mRNA expression levels of these factors (data not shown). Following the combined treatments of CSE together with E_2_ and MPA, the *IGFBP‐1* mRNA expression levels significantly increased at 0.01% and 0.025% CSE concentrations compared to control. However, these levels were significantly suppressed at 0.25% CSE concentration, with a decreased trend in expression observed after treatment with 0.1% CSE (*P = *.07; Figure 8A). As shown in Figure 8B,C, exposure to E_2_ and MPA resulted in significantly increased levels of *HAND2* and *IL‐15* mRNA expression at 0.01% and 0.025% CSE concentrations, which were significantly suppressed at 0.1% and 0.25% CSE concentrations. These results revealed that differing concentrations of CSE exerted similar expression patterns for PRL and IGFBP‐1. We also evaluated FGF9 expression, which is suppressed following exposure to E_2_ and MPA, and is the downstream target of HAND2 in ESCs.^19^ Our results showed that E_2_ and MPA treatment attenuated *FGF9* mRNA expression; however, no significant differences were noted, regardless of CSE concentration (Figure 8D).

## 4 DISCUSSION

In this study, we have provided new insights into the effects of CSE on the regulatory mechanisms of angiogenesis and decidualization in ESCs. CSE induced *VEGF* mRNA expression levels in a dose‐dependent manner, as well as HIF‐1α protein levels in ESCs. These results suggest that HIF‐1α activation by CSE enhances *VEGF* mRNA expression in ESCs. Additionally, to the best of our knowledge, our study is the first to demonstrate that CSE induces differential effects on PRL and the other decidual specific factors evaluated depending on the concentration of CSE tested. In brief, low concentrations of CSE increased the expression levels of PRL and other decidual specific factors, whereas high concentrations of CSE suppressed the expression levels. Based on our findings, CSE affects the production of angiogenic factors, HIF‐1α activation, and decidualization in ESCs.

The toxicity test for cigarette smoke is commonly used in animal or cell experiments, and CSE is widely employed in in vitro models.^28^ Earlier studies report that 1% CSE approximately corresponds to exposures associated with smoking slightly less than two packs of cigarettes per day in pulmonary artery endothelial cells.^29^, ^30^ Based on these studies, we propose that 0.25% CSE concentration closely represents exposure faced by average smokers. However, whether CSE concentration is similar between pulmonary artery endothelial cells, which directly absorb cigarette smoke, and ESCs is unknown.

Concurring with our results, nicotine in cigarette smoke has been shown to induce VEGF expression in human ESCs, regardless of ovarian steroid hormones.^31^ VEGF is essential for implantation and placentation ^32^; however, higher levels of VEGF may disrupt normal angiogenesis through an overstimulation of blood vessels leading to disturbed vascular architecture.^33^, ^34^ Taken together, it is important to consider the influence of these results of the present study on implantation, as the effects of CSE on angiogenesis may adversely affect the establishment of pregnancy.

In the promoters encoding *VEGF* genes, HIF‐1α has been shown to directly bind to the hypoxia response element. In the human endometrium, HIF‐1α is expressed with increasing intensity from the premenstrual to the menstrual phase.^35^ Previous studies showed that both HIF‐1α and HIF‐2α have a functional role in embryo implantation.^36^, ^37^ HIF‐1α is a transcription factor known to play a critical role in the cellular response to hypoxia. However, under normoxic conditions, HIF‐1α is synthesized in a kinase inhibitor‐sensitive manner via PI3K, Akt, and mTOR pathways.^38^, ^39^ In fact, CSE is reported to activate HIF‐1α under normoxic conditions both in vitro and in vivo.^40^ In human lung adenocarcinoma A549 cells, CSE induced the expression of VEGF via HIF‐1 activation in a reactive oxygen species (ROS)‐dependent manner.^40^ HIF‐1α also is shown to be involved in the induction of VEGF in human ESCs and directly binds to the promoters of the genes encoding VEGF.^41^ Our results concur with findings from earlier studies that showed CSE‐mediated induction of VEGF expression and HIF‐1α accumulation in ESCs under normoxia conditions in the absence of E_2_ and MPA.

GLUT1 has a HIF‐1α binding sequence in its promoter.^27^ In this study, CSE simultaneously increased *GLUT1* mRNA expression and the accumulation of HIF‐1α. We recently demonstrated that echinomycin, a small molecule inhibitor of HIF‐1α activity, substantially reduced GLUT1 expression under hypoxia in ESCs, suggesting that HIF‐1α plays a major role in regulating GLUT1 expression. The physiological role of GLUT1 is that in a hypoxic environment such as menstrual and implantation periods, ESCs increase extracellular glucose uptake and enhance glycolysis, thereby obtaining energy.^42^ Herein, these findings suggest that CSE‐induced HIF‐1α plays a key role in the regulation of GLUT1 and VEGF expression.

The observation from this study that 0.25% CSE significantly decreased ANGPT1 expression, but had no effect on ANPGT2 expression, is consistent with previous data showing that hypoxia reduced the mRNA expression and protein production of ANGPT1 in ESCs, whereas those of ANGPT2 remained unaffected.^9^ Recent studies have reported that ROS decreases the expression of *ANGPT1* mRNA and proteins in cultured human ESCs.^43^ CSE‐induced ROS may play a role in the reduction of ANGPT1 expression. Interestingly, VEGF expression in ESCs is affected by low concentrations of CSE, whereas ANGPT1 expression is modulated only at 0.25% CSE concentration. Further research is needed to clarify the different mechanisms that regulate VEGF and ANGPT1 expression in response to CSE.

We demonstrated that CSE affects the expression of decidual specific factors. The levels of *PRL* mRNA and PRL protein were elevated after treatment with 0.01% and 0.025% CSE compared to control, but expression levels were then suppressed at 0.1% and 0.25% CSE concentrations in the presence of E_2_ and MPA. However, without E_2_ and MPA, CSE had no effect on the levels of PRL expression, suggesting that CSE affects PRL levels during decidualization. The observed morphological changes correlated with the changes in PRL levels after exposure to different CSE concentrations. Cadmium, one of the major contaminants of cigarette smoke, markedly elevates PRL levels and stimulates decidualization in ESCs.^44^ Another study also showed that CSE increased the expression of endometrial homeobox 10 and the progesterone receptor and promoted early decidualization in immortalized endometrial cell lines of ESC.^45^ In this study, treatment with 0.01% and 0.025% CSE also upregulated *IGFBP‐1* mRNA in ESCs. The time point of IGFBP‐1 secretion, which is about 10 d after the luteinizing hormone peak in vivo, is relevant for a marked reduction in endometrial receptivity and a rapidly increasing risk of implantation failure.^8^ Therefore, CSE may affect the endometrial receptivity by upregulating IGFBP‐1.

There are very few reports that CSE suppresses decidualization. To the best of our knowledge, there is one report using female rat models showing the potential effects of nicotine on endometrial decidualization by assessing by the weight of the uterus after mechanically induced decidualization. The authors concluded that there was an adverse effect on the decidualization process resulting in a lower uterus weight after nicotine administration.^46^ In the present study, we showed that CSE resulted in differential gene expressions depending on the concentration tested. This may be attributed to concentration differences of the principal substances within CSE, at the different concentrations of the CSE extract solution. Further studies are warranted to determine the influence of the main individual components of CSE on gene expressions.

As previously mentioned, HAND2 is a transcription factor and progestin‐induced HAND2 plays a key role in the regulation of PRL and IGFBP‐1 expression in ESCs.^47^ It is also reported that HAND2 enhanced IL‐15 and suppressed FGF9 in ESCs.^17^, ^19^ Our study demonstrates that CSE may exert effects on the expression of PRL, IGFBP‐1, and IL‐15 via HAND2. In contrast, E_2_ and MPA attenuated the *FGF9* mRNA levels, but CSE treatment alone did not affect expression.

There are several limitations to our study that need to be considered. First, the effects of CSE on the mRNA levels of the decidual specific factors were measured; however, future studies need to confirm the translation of the mRNA into protein. Additionally, all patients from whom the tissue samples were obtained were non‐smokers. Thus, further evaluation of the effects of CSE on ESCs from non‐smokers and smokers is warranted. Moreover, the impact of smoking on endometrial maturation including angiogenesis and decidualization needs to be corroborated in vivo. Another limitation of this study is the low sample number. Increasing the sample number could provide stronger statistical verification of our findings. The final limitation is the concentration and composition of CSE. As mentioned above, previous studies have reported the concentrations of CSE in smokers,^27^, ^28^ but these findings may or may not be applicable to our study, and hence, further studies on the tissue concentrations of CSE in ESCs are needed. Moreover, cigarette smoke contains more than 4000 toxic compounds,^48^ of which one or more can induce or reduce the expression of genes that play a role in angiogenesis and decidualization of ESCs. Isolation and characterization of these compounds may help clarify the paradoxical observation that CSE caused differential gene expressions depending on the concentration tested and also lead to the development of therapeutic strategies.

Collectively, our study demonstrates that CSE above 0.025% enhances the expression of angiogenic factors, and CSE above 0.01% affects the expression of decidual specific factors in ESCs in the presence of E_2_ and MPA. These results highlight that exposure to even a small amount of cigarette smoke could affect ESCs. Our study provides a novel insight in that cigarette smoke directly affects the human endometrium. Moreover, our results support epidemiological studies that cigarette smoke has an adverse effect on reproductive outcome.

## Conflict of interest

The authors declare no conflict of interest.

## Human rights, informed consent, and ethical approval

All the procedures were followed in accordance with the ethical standards of the institutional ethical committee on human experimentation (institutional and national) and with the Helsinki Declaration of 1964 and its later amendments. In this study, informed consent was obtained from all the patients who underwent hysterectomy. This study was approved by the Institutional Review Board at Kansai Medical University.

## Animal studies

This article does not contain any studies with human and animal subjects performed by the any of the authors.
